# Expression of tissue levels of matrix metalloproteinases and tissue inhibitors of metalloproteinases in renal cell carcinoma

**DOI:** 10.1186/1477-7819-11-1

**Published:** 2013-01-03

**Authors:** Zhen-kui Qiao, Yan-long Li, Hong-tao Lu, Ke-liang Wang, Wan-hai Xu

**Affiliations:** 1 Department of Urology, the Fourth Affiliated Hospital of Harbin Medical University, 37 Yiyuan Road, Harbin, 150001, China

**Keywords:** Matrix metalloproteinase, Tissue inhibitor of metalloproteinase, Semi-quantitative RT-PCR, Renal cell carcinoma

## Abstract

**Background:**

Matrix metalloproteinases (MMPs) are one of the major classes of proteolytic enzymes involved in tumor invasion and metastasis and are inhibited by naturally occurring tissue inhibitors of metalloproteinases (TIMPs). {AU Query: Please verify that corrections made to previous sentence did not alter intended meaning}. In this study, we examined the expression of MMP-2, MMP-9, membrane-type 1 (MT1)-MMP, TIMP-1, and TIMP-2 in renal tissue samples of renal cell cancer and examined the correlation between their expression and clinicopathological parameters.

**Methods:**

Renal tissue samples from 76 patients with renal cell carcinoma were available for this study. To determine the expression of MMP-2, MMP-9, MT1-MMP, TIMP-1, and TIMP-2, semi-quantitative reverse transcriptase-polymerase chain reaction (RT-PCR) was carried out on tumor and normal tissues.

**Results:**

Mean MMP-2, MMP-9, MT1-MMP, TIMP-1, and TIMP-2 mRNA expression in the renal cell carcinomas was significantly higher than in the normal renal tissue (*P* <0.05). The RT-PCR data of MMP-2, MMP-9, MT1-MMP, TIMP-1, and TIMP-2 did not show any significant correlation with tumor type or pathologic grade of renal cell carcinoma. MMP-2, MMP-9 and MT1-MMP mRNA expression increased significantly with the TNM stage of the tumor.

**Conclusions:**

Mean MMP-2, MMP-9, MT1-MMP, TIMP-1, and TIMP-2 mRNA expression in the renal cell carcinomas was significantly higher than in the normal renal tissue.

## Background

Metastasis of renal cell carcinoma cells depends on some factors that are only partly understood: proteolysis, cellular attachment, angiogenesis, migration through the barrier into secondary sites, and colonization and proliferation in distant organs [1]. Proteolytic degradation of the basement membrane is a fundamental aspect of cancer development and a key event in the regulation of tumor proliferation and metastasis [2]. The invasion of the basement membrane proceeds through a series of discrete steps [3]. The matrix degradation in the basement membrane is closely related to activities of various subtypes of matrix metalloproteinases (MMPs) and the corresponding tissue inhibitors of matrix metalloproteinase (TIMPs).

MMPs are secreted as inactive pre-enzymes and are transformed into active forms after cleavage of a propeptide domain of the molecule [4]. On the basis of their structure, cell localization, and substrate specificity, the more than 20 human MMPs are divided into several groups such as collagenases, gelatinases, stromelysins, and membrane-type MMPs (MT-MMPs) [5]. Among the MMPs, MMP-2 and MMP-9 have been the focus of attention in connection with cancer metastasis because of their ability to degrade type IV collagen, a major constituent of the vascular basement membrane [6]. MT1-MMP was the first member of the MT-MMP family to be discovered, since it is tethered to the plasma membrane [7]. The expression of MT1-MMP has been thought to initiate multiple protein cascades on the cell surface [8].

MMP-2 is an important enzyme of the MMP family, which is able to degrade collagen IV, a basic component of constitutive basement membranes [9]. The activation and enzymatic activity of MMP-2 is regulated by TIMP-2 [10]. MMP-2 has been considered essential for metastasizing tumor cells. In this context, evaluation of MMP-2 expression in lung and colon cancer appears to be a useful prognostic indicator [11,12]. Recent studies have reported an alternative function of TIMP-1, that is, as a growth factor; it is highly homologous with erythroid-potentiating activity, which is an autocrine growth factor for the erythroid leukemia cell line K562 [13]. Moreover, TIMP-1 also shares homology with a fibroblast elongation factor that is secreted from colon carcinoma cells and that stimulates tumor cell proliferation [14]. TIMP-1 RNA levels are higher in primary colorectal carcinomas with distant metastasis than in those without metastasis [15], and the expression of TIMPs increases with the advance of the neoplastic process [16].

The expression and involvement of several MMPs and TIMPs in human renal cell carcinoma have been determined in several studies. However, the studies showed relatively conflicting results about their contribution to the clinicopathological findings and prognosis of the patients with renal cell carcinoma. In the present study, we examined the expression of MMP-2, MMP-9, MT1-MMP, TIMP-1, and TIMP-2 mRNA in human renal cell carcinoma tissues by a reverse transcriptase-polymerase chain reaction (RT-PCR) assay and examined the correlation between their expression and clinicopathological parameters.

## Methods

### Patients

Renal tissue samples from 76 patients (45 men and 31 women) with renal cell carcinoma, for whom clinical and histopathological data concerning the patients and carcinomas were available, were analyzed. Renal tissue samples were obtained from cancerous and non-cancerous parts of the same kidney, which had been surgically removed by radical nephrectomy. Tumors were graded according to Fuhrman’s system [17] and staged according to TNM criteria [18]. For statistical evaluations, grade 1 and 2 tumors were considered as low grade and grade 3 and 4 as high grade. Similarly, stage 3 and 4 tumors were considered in the advanced stage category. Four patients at the time of diagnosis {AU Query: Is this an acceptable term? Or do you mean at the time of diagnosis?} already had a metastatic tumor. The tumor and normal tissue samples were snap-frozen in liquid nitrogen immediately after surgical removal and stored at −80°C for RNA extraction. Mean age of the patients was 61.3 ± 13.5 years, ranging from 28 to 84 years. Informed consent had been obtained, and the Ethics Committee of Harbin Medical University approved this study.

### RNA extraction and reverse transcriptase polymerase chain reaction

Total RNA was extracted from renal tissue samples using ISOGEN (Nippon Gene, Toyama, Japan). The amount and purity of the extracted RNA was determined by spectrophotometry. The cDNA was synthesized with 5 μg of total RNA and oligo dT primer. The cDNA was amplified using primers specific for the MMP-2, MMP-9, MT1-MMP, TIMP-1 and TIMP-2 genes, or for the β-actin gene, which was used as a control (Table 1). PCR conditions for MMP-2 and β-actin amplification were 30 cycles at 94°C for 30 seconds, 62°C for 30 seconds, and 72°C for 30 seconds, followed by incubation at 72°C for 5 minutes. PCR conditions for MT1-MMP amplification were 30 cycles at 94°C for 1 minute, 60°C for 1 minute, and 72°C for 1 minute, followed by incubation at 72°C for 5 minutes. PCR conditions for MMP-9, TIMP-1 and TIMP-2 amplification were 30 cycles of 94°C for 1 minute, 56°C for 30 seconds, and 72°C for 1 minute, followed by incubation at 72°C for 5 minutes. The PCR mixture was amplified using a GeneAmp PCR System 9600 (PerkinElmer, Wellesley, MA, USA).

**Table 1:** Oligonucleotide primers sequences for mRNA amplification

	**Direction**	**Primer sequence**	**Fragment size (bp)**
MMP-2	Sense	ACCTGGATGCCGTCGTGGAC	448
	Antisense	TGTGGCAGCACCAGGGCAGC	
MMP-9	Sense	CGCTGGGCTTAGATCATTCC	460
	Antisense	TTGTCGGCGATAAGGAAGG	
MT1-MMP	Sense	TGACGGGAACTTTGACACC	262
	Antisense	CAGCTCCTTAATGTGCTTGG	
TIMP-1	Sense	GGGCTTCACCAAGACCTA	280
	Antisense	GAAGAAAGATGGGAGTGGG	
TIMP-2	Sense	CCAAAGCGGTCAGTGAGA	421
	Antisense	TGGTGCCCGTTGATGTTC	
β-actin	Sense	AAGATGACCCAGATCATGTTTGAG	648
	Antisense	AGGAGGAGCAATGATCTTGATCTT	

Amplified products (10 μl) were identified by electrophoresis of the PCR products on a 1.5% agarose gel containing ethidium bromide and ultraviolet illumination. The housekeeping gene β-actin was used as a control and for semi-quantitative analysis of the MMP-2, MMP-9, MT1-MMP, TIMP-1, and TIMP-2. A negative control, with H2O instead of cDNA, was also used. The levels of gene transcripts were quantified as the ratio of the intensity of the target gene to the intensity of β-actin.

### Statistical analysis

The results were expressed as means ± the standard deviation. The expression of MMP-2, MMP-9, MT1-MMP, TIMP-1, and TIMP-2 were performed with Student’s *t*-test. The association between the clinicopathological variables and the expression of MMP-2, MMP-9, MT1-MMP, TIMP-1, and TIMP-2 was also analyzed using Student’s *t*-test. The accepted level of significance was *P* <0.05. All data analysis was performed using the SPSS for Windows, Version 10.0 software package (SPSS Inc, Chicago, IL).

## Results

Of the 76 renal cell carcinomas, there were 59 clear cell, 11 papillary, 4 chromophobe, and 2 sarcomatoid carcinomas. (Table 2) There were 45 male (59.2%) and 31 female (40.8%) patients aged from 28 to 84 years with a mean age of 61.3 ± 13.5 years. The tumors were categorized as grade 1 and 2 in 57 (75%) cases and grade 3 and 4 in 19 (25%) cases; and stage 1 and 2 in 52 (68%) cases and stage 3 and 4 in 24 (32%) cases.

**Table 2:** Clinicopathological features of 76 renal cell carcinomas

**Variables**	**Renal cell carcinoma (n = 76)**
Age (mean, years)	61.3 ± 13.5
Sex	
Male	45
Female	31
Tumor type	
Clear cell	59
Papillary	11
Chromophobe	4
Sarcomatoid carcinoma	2
Pathologic grade	
Grade 1 and 2	57
Grade 3 and 4	19
Tumor stage	
Stage 1 and 2	52
Stage 3 and 4	24

**Figure 1:**
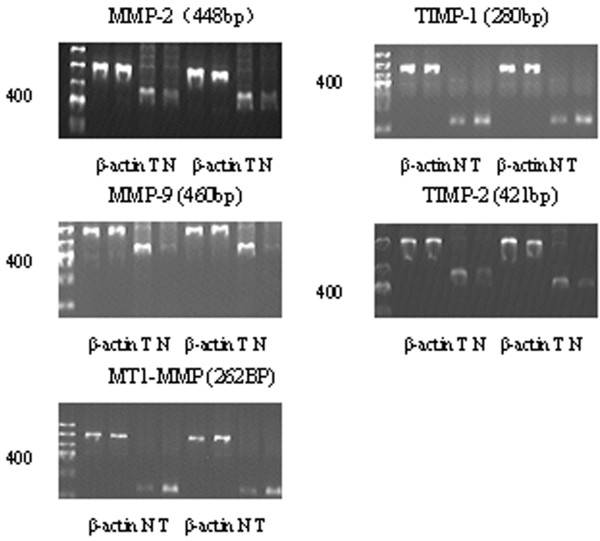
**Reverse transcriptase polymerase chain reaction analysis of mRNA expression for proteolytic enzymes in normal and tumor tissues. **N, normal tissue; T, tumor tissue; MMP, matrix metalloproteinase; MT1-MMP, membrane-type 1 matrix metalloproteinase; TIMP, tissue inhibitor of matrix metalloproteinase.

Expression of MMPs and TIMPs could be detected in all renal tissue samples, and their levels varied among cases. The mRNA expression of the proteolytic enzymes MMP-2, MMP-9, MT1-MMP, TIMP-1, and TIMP-2 is shown in Figure 1. A comparison of the relative amounts of MMP-2, MMP-9, MT1-MMP, TIMP-1, and TIMP-2 mRNAs in renal cell carcinomas and normal renal tissue is shown in Table 3. Mean MMP-2, MMP-9, MT1-MMP, TIMP-1, and TIMP-2 mRNA expression in the renal cell carcinomas was significantly higher than in the normal renal tissue (*P* <0.05).

**Table 3:** Expression of matrix metalloproteinases (MMPs) and tissue inhibitors of metalloproteinases (TIMPs)

	**Tumor tissue**	**Normal tissue**	***P *value**
MMP-2	0.732 ± 0.067	0.342 ± 0.021	<0.05
MMP-9	0.701 ± 0.089	0.238 ± 0.019	<0.01
MT1-MMP	0.460 ± 0.034	0.191 ± 0.022	<0.01
TIMP-1	0.375 ± 0.042	0.153 ± 0.020	<0.05
TIMP-2	0.386 ± 0.015	0.234 ± 0.013	<0.05

In terms of pathologic grade of renal cell carcinoma, the differences in MMP-2, MMP-9, MT1-MMP, TIMP-1, and TIMP-2 mRNA expression levels were not significant. Similarly, MMP-2, MMP-9, MT1-MMP, TIMP-1, and TIMP-2 mRNA expression did not differ significantly in relation to tumor type of renal cell carcinoma (Table 4). In terms of tumor stage of renal cell carcinoma, the differences in MMP-2, MMP-9 and MT1-MMP mRNA expression levels were significant (Table 5). MMP-2, MMP-9 and MT1-MMP mRNA expression increased significantly with the TNM stage of the tumor.

**Table 4:** Expression of matrix metalloproteinases (MMPs) and tissue inhibitors of metalloproteinases (TIMPs) by tumor type and pathologic grade

	**Tumor**	**Type**		**Pathologic**	**Grade**	
	**Clear cell**	**Non-clear cell**	***P *value**	**High**	**Low**	***P *value**
MMP-2	0.741 ± 0.064	0.699 ± 0.078	NS	0.722 ± 0.056	0.796 ± 0.095	NS
MMP-9	0.730 ± 0.086	0.623 ± 0.069	NS	0.685 ± 0.062	0.749 ± 0.090	NS
MT1-MMP	0.478 ± 0.036	0.393 ± 0.029	NS	0.451 ± 0.042	0.485 ± 0.031	NS
TIMP-1	0.369 ± 0.051	0.390 ± 0.034	NS	0.394 ± 0.046	0.319 ± 0.040	NS
TIMP-2	0.388 ± 0.029	0.379 ± 0.023	NS	0.383 ± 0.014	0.395 ± 0.017	NS

NS, not significant.

**Table 5:** Expression of matrix metalloproteinases (MMPs) and tissue inhibitors of metalloproteinases (TIMPs) by tumor stage

	**Tumor**	**Stage**	
	**Low stage**	**Advanced stage**	***P *value**
MMP-2	0.652 ± 0.043	0.901 ± 0.068	*P* <0.05
MMP-9	0.604 ± 0.035	0.923 ± 0.077	*P* <0.05
MT1-MMP	0.320 ± 0.038	0.749 ± 0.080	*P* <0.05
TIMP-1	0.353 ± 0.046	0.412 ± 0.051	NS
TIMP-2	0.373 ± 0.029	0.402 ± 0.042	NS

NS, not significant.

## Discussion

Tumor growth, invasion, and metastasis is a multistep process that is facilitated by the proteolytic degradation of components of the extracellular matrix (ECM) and basement membrane. The role of MMPs in this process has been firmly established based on numerous previously published experimental and clinical studies. In the present study, mean MMP-2, MMP-9, MT1-MMP, TIMP-1, and TIMP-2 mRNA expression in the renal cell carcinomas was significantly higher than in the normal renal tissue (*P* <0.05). MMP-2, MMP-9, MT1-MMP, TIMP-1, and TIMP-2 mRNA expression did not differ significantly in relation to tumor type or pathologic grade of renal cell carcinoma. MMP-2, MMP-9 and MT1-MMP mRNA expression increased significantly with the TNM stage of the tumor.

Several studies have suggested that the role of MMP-2 and MMP-9 in the digestion of basement membrane type IV collagen is an important mechanism for vessel invasion and metastasis [6,19]. Because of its ability to degrade the basement membrane, MMP-2 has been postulated to be a potential marker of tumor progression and prognosis. Gohji *et al*. [20] reported increased serum levels of MMP2 in patients with urothelial cancer that correlated with disease progression and poor outcome. Kugler *et al*. [21] analyzed MMP2, MMP9, TIMP1, and TIMP2 in 17 renal cell carcinomas by PCR and demonstrated a strong correlation between increased gene expression and tumor stage. Some *in vivo* and *in vitro* experiments showed that MMP levels were related to the invading and metastatic potential of colorectal cancer. Sier *et al*. [19] demonstrated that higher tissue levels of total MMPs and the pro-forms of MMP-2 and MMP-9, as well as the active form of MMP-2 indicated a poor prognosis in patients with gastric cancer. Some authors reported that the matrix-degrading activity of MMP-9 is nearly 25 times that of MMP-2, and that MMP-9 is more important for the metastatic potential of carcinoma than MMP-2 [22,23]. Furthermore, MMP-9 expression by immunohistochemistry has been found to be significantly correlated with poor prognosis in renal cell carcinoma [24]. In contrast, another study showed no significant correlation between MMP-9 mRNA expression and the prognosis of patients with hepatocellular cancer. Baseline levels of MMP-9 warrant further study as predictive markers of sunitinib activity in metastatic renal-cell carcinoma (MRCC) [25]. Porta *et al*. [26] reported that serum levels of VEGF and NGAL are significant predictors of progression-free survival in patients with renal cell carcinoma treated with sunitinib. In our study, mean MMP-2 and MMP-9 mRNA expression in the renal cell carcinomas was significantly higher than in the normal renal tissue (*P* <0.05), the levels of MMP-2 and MMP-9 mRNA expression were not correlated with tumor type or pathologic grade of renal cell carcinoma. MMP-2 and MMP-9 mRNA expression increased significantly with the TNM stage of the tumor.

MT1-MMP, the first member of a more recently established group of MMPs containing a membrane-spanning sequence, has been shown to have an important role in MMP-2 activation in cell membranes, and its overexpression seems to have a significant effect on tumor growth. Expression of MT1-MMP mRNA tends to be associated with a lower degree of differentiation in hepatocellular cancer and has a strong statistical association with poor prognosis [27]. Moreover, a similar tendency was also observed in relation to pancreatic adenocarcinomas, but the association did not reach statistical significance [27]. In addition, Kitagawa *et al*. [28] showed that MT1-MMP in particular was associated with invasiveness of renal cell carcinoma. In this study, mean MT1-MMP mRNA expression in the renal cell carcinomas was significantly higher than in the normal renal tissue (*P* <0.01), and the levels of MT1-MMP mRNA expression were not correlated with tumor type or pathologic grade of renal cell carcinoma. MT1-MMP mRNA expression increased significantly with the TNM stage of the tumor.

In the context of tumor invasion, the original understanding of TIMPs was that of an inhibitor of MMPs, thus serving as anti-invasive/anti-metastatic agents. {AU Query: Please verify that changes made to the previous sentence did not alter intended meaning.}TIMPs have been reported to be negative regulators of MMPs in mouse tumor models and in humans, *in vitro* and *in vivo*[29]. However, in another study using clinical samples, the expression of TIMP mRNA was higher in carcinoma tissues. In studies of various types of carcinoma, such as head and neck, colorectal, stomach, and pancreatic carcinomas, both MMPs and TIMPs were found to correlate with increased metastatic and invasive potential of tumor cells [15,30]. Furthermore, TIMP-1 and TIMP-2 expression by immunohistochemistry has been found to be significantly correlated with poor prognosis in renal cell carcinoma [24]. In the present study, mean TIMP mRNA expression in the renal cell carcinomas was significantly higher than in the normal renal tissue (*P* <0.05). *et al*. [21] and Kallakury *et al*. [24].

Although both neoplastic and normal cells produce MMPs and other proteinases, only malignant cells are invasive [31]. Therefore, it is more likely that the control of MMP activity by specific inhibitors is one cause of the different functions of these enzymes in neoplastic and normal tissues. Ko *et al*. [32] has reported that TIMP-2 is inversely correlated with nodal metastasis and that TIMP-2 expression is stronger in early gastric cancer than in advanced gastric cancer, indicating that TIMP-2 may play an important role in protection against MMPs. However, another study showed that the expression of TIMP-2 was not associated with variable clinicopathological parameters, and that the status of TIMP-2 expression was variable in many types of cancer tissues. In our study, TIMP-1, and TIMP-2 mRNA expression did not differ significantly in relation to tumor type, pathologic grade or tumor stage of renal cell carcinoma.

## Conclusions

In summary, mean MMP-2, MMP-9, MT1-MMP, TIMP-1, and TIMP-2 mRNA expression in the renal cell carcinomas was significantly higher than in the normal renal tissue (*P* <0.05). MMP-2, MMP-9, MT1-MMP, TIMP-1, and TIMP-2 mRNA expression did not differ significantly in relation to tumor type or pathologic grade of renal cell carcinoma. MMP-2, MMP-9 and MT1-MMP mRNA expression increased significantly with the TNM stage of the tumor.

## Abbreviations

ECM: extracellular matrix; MMPs: matrix metalloproteinases; MRCC: metastatic renal-cell carcinoma; MT1: membrane-type 1; MT-MMPs: membrane-type MMPs; TIMPs: tissue inhibitors of metalloproteinases; RT-PCR: reverse transcription polymerase chain reaction.

## Competing interests

The authors declare that they have no competing interests.

## Authors’ contributions

QZ participated in the design of the study and drafted the manuscript. XW conceived the study, participated in the design of the study and helped to draft the manuscript. LY participated in acquisition of data and performed the statistical analysis. LH participated in acquisition of data and performed the statistical analysis. WK participated in its design and helped to draft the manuscript. All authors read and approved the final manuscript.
